# Identifying Patient-Specific Epstein-Barr Nuclear Antigen-1 Genetic Variation and Potential Autoreactive Targets Relevant to Multiple Sclerosis Pathogenesis

**DOI:** 10.1371/journal.pone.0147567

**Published:** 2016-02-05

**Authors:** Monika Tschochner, Shay Leary, Don Cooper, Kaija Strautins, Abha Chopra, Hayley Clark, Linda Choo, David Dunn, Ian James, William M. Carroll, Allan G. Kermode, David Nolan

**Affiliations:** 1 Institute for Immunology & Infectious Diseases, Murdoch University, Perth, Western Australia, Australia; 2 Department of Neurology, Sir Charles Gairdner Hospital, Perth, Western Australia, Australia; 3 Centre for Neuromuscular and Neurological Disorders, Australian Neuromuscular Research Institute, Nedlands, Western Australia, Australia; 4 Department of Clinical Immunology, Royal Perth Hospital, Perth, Western Australia, Australia; University of Sussex, UNITED KINGDOM

## Abstract

**Background:**

Epstein-Barr virus (EBV) infection represents a major environmental risk factor for multiple sclerosis (MS), with evidence of selective expansion of Epstein-Barr Nuclear Antigen-1 (EBNA1)-specific CD4+ T cells that cross-recognize MS-associated myelin antigens in MS patients. HLA-DRB1*15-restricted antigen presentation also appears to determine susceptibility given its role as a dominant risk allele. In this study, we have utilised standard and next-generation sequencing techniques to investigate EBNA-1 sequence variation and its relationship to HLA-DR15 binding affinity, as well as examining potential cross-reactive immune targets within the central nervous system proteome.

**Methods:**

Sanger sequencing was performed on DNA isolated from peripheral blood samples from 73 Western Australian MS cases, without requirement for primary culture, with additional FLX 454 Roche sequencing in 23 samples to identify low-frequency variants. Patient-derived viral sequences were used to predict HLA-DRB1*1501 epitopes (NetMHCII, NetMHCIIpan) and candidates were evaluated for cross recognition with human brain proteins.

**Results:**

EBNA-1 sequence variation was limited, with no evidence of multiple viral strains and only low levels of variation identified by FLX technology (8.3% nucleotide positions at a 1% cut-off). In silico epitope mapping revealed two known HLA-DRB1*1501-restricted epitopes (‘AEG’: aa 481–496 and ‘MVF’: aa 562–577), and two putative epitopes between positions 502–543. We identified potential cross-reactive targets involving a number of major myelin antigens including experimentally confirmed HLA-DRB1*15-restricted epitopes as well as novel candidate antigens within myelin and paranodal assembly proteins that may be relevant to MS pathogenesis.

**Conclusions:**

This study demonstrates the feasibility of obtaining autologous EBNA-1 sequences directly from buffy coat samples, and confirms divergence of these sequences from standard laboratory strains. This approach has identified a number of immunogenic regions of EBNA-1 as well as known and novel targets for autoreactive HLA-DRB1*15-restricted T cells within the central nervous system that could arise as a result of cross-reactivity with EBNA-1-specific immune responses.

## Introduction

Epstein-Barr virus (EBV) is the only human-adapted member of the *Lymphocryptovirus* genus, belonging to a lineage of Old World primate gamma-1 herpesviruses that was transferred to a hominid ancestor approximately twelve million years ago, and which is now responsible for near-universal and lifelong human infection [1,2]. Viral transmission is generally via saliva, with evidence that age of infection is associated with cultural and socioeconomic factors [3]. Uniquely, chronic infection is established within ‘immortalised’ B-lymphocytes that are transformed by an array of viral proteins that functionally mimic host proteins to create long-lived memory cells [4,5]. Viral persistence is then promoted through mechanisms that reduce antigen presentation to the adaptive immune system [6], including the involvement of latency programs that limit viral protein expression to a minimal subset critical for replication; most notably Epstein-Barr Nuclear Antigen-1 (EBNA-1), which maintains host chromosomal attachment of viral episomal DNA thus linking viral and cellular replication cycles [7].

These mechanisms of viral persistence would predict limited viral sequence diversity, in keeping with the relatively slow evolutionary rate of EBV and other gamma-1 herpesviruses [2] and evidence of geographically-defined viral subtypes [8]. Nevertheless, evidence of diversifying selection involving latency genes including EBNA-1 has been identified [9], including preferential variation within human leukocyte antigen (HLA) binding sites (viral epitopes) suggesting that antigen presentation can promote HLA-specific viral escape mutations [10,11]. Thus, EBNA-1 is not immunologically ‘silent’ as once thought [12] but is an antigenic target for both CD4 and CD8 T-cell responses [12–14] as well as antibodies [15], in keeping with finely tuned immune surveillance mechanisms that generally maintain persistent but stable cycles of EBV infection involving both epithelial and B-lymphocyte compartments [5]. Within this paradigm, mechanisms of viral antigen display [13,14] and the general hierarchy of EBV-specific immune responses including regulatory as well as effector T cell responses are being examined [14,16–18]. These have particular relevance to the therapeutic application of EBV-specific T-cell adoptive immunotherapy against EBV-related malignancies including Burkitt’s and Hodgkin’s lymphoma and nasopharyngeal carcinoma [19], now supported by positive findings in clinical trials [20,21]. This strategy is underpinned by knowledge of EBV sequence diversity in tissue samples [9,22,23] and its utilisation to predict viral epitope targets [24].

Our own investigations have focused on multiple sclerosis, an inflammatory demyelinating disease of the central nervous system that often leads to neurodegeneration and long-term disability despite current treatment strategies [25]. While a comprehensive explanation of multiple sclerosis pathogenesis remains incomplete, it is clear that the major component of genetic risk is associated with the HLA-DR locus [26–29], thus implicating HLA-restricted antigen binding and presentation [30], as well as genetic determinants that predominantly relate to T-cell activation [29]. Several lines of evidence link Epstein-Barr virus-specific immunity to multiple sclerosis risk. Both serological [27,31,32] and CD4 T cell responses [33] directed against EBNA-1 have been associated with multiple sclerosis, with further evidence that EBNA-1-specific antibodies differentiate disease-discordant identical twins [34]. Several groups have demonstrated higher EBV seroprevalence in MS patients compared to controls and it has further been demonstrated that EBV infection late in life, in particular if manifested as infectious mononucleosis, increases a person’s MS risk [27, 35, 36]. A recent study has also explored the use of Epstein-Barr virus-specific adoptive immunotherapy for progressive multiple sclerosis, with promising preliminary results [37]. Further observations include that cerebrospinal fluid oligoclonal bands that are a hallmark of MS specifically can target EBNA-1 [38] and one group has additionally identified the presence of EBV-infected B cells within white matter MS lesions at all disease stages [39], although this result has not been replicated in other studies [40].

In this study, we have utilised DNA obtained from buffy coat samples of patients with multiple sclerosis to analyse EBNA-1 sequence variation using both Sanger and FLX ‘next-generation’ sequencing technologies, without any requirement for primary culture techniques or the creation of cell lines through *ex vivo* EBV transformation. We have then sought to identify potential HLA-DRB1*1501-restricted viral epitopes within the EBNA-1 protein sequence using standard HLA binding algorithms [41], and investigated potential homology with similarly HLA-restricted antigens in a dataset of human central nervous system proteins [42]. The results of this study, which follow from our previous investigations of the contributions made by HLA alleles and Epstein-Barr virus immunity to multiple sclerosis risk [27,28], highlight the divergence of autologous ‘wild-type’ EBNA-1 sequences from those of laboratory strains commonly used for experimental purposes, and suggest possible avenues of investigation that acknowledge both host and viral genetic diversity in higher-resolution analyses of the role of host-pathogenic interactions in autoimmunity [30,43,44].

## Materials and Methods

### Research participants

A total of 79 study participants in the Perth Demyelinating Disease Database (PDDD) were included in the study. The study protocol was approved by the Sir Charles Gairdner Hospital Human Research Ethics Committee, and written informed consent was obtained from all participants.

### DNA extraction

DNA was isolated from buffy coats (stored at -80°C) using an automated robotic setup using Genfind according to the manufacturer’s instructions. Briefly, 100ul of buffy coat were lysed and Proteinase K added to rupture cell membranes and digest protein. DNA was then immobilized on magnetic particles by the addition of a magnetic bead binding reagent. DNA was separated from contaminants using a magnetic field and washing steps. DNA was eluted in 125μl from the magnetic particles. A minority of samples were manually extracted using Qiagen with the provided protocol. 200μl of buffy coat were lysed and Proteinase K added to remove protein and other contaminants. DNA was absorbed on to the silica-gel membrane during centrifugation of columns and then washed twice to ensure complete removal of any residual contaminants. Finally, samples are recovered from the membrane using 200μL elution buffer. Concentration of all eluted samples was determined using Nanodrop and 1 μl of each sample was loaded on a 1% agarose gel to test for presence and integrity of DNA. All samples were stored at 4°C until further use.

### HLA typing

PCR and sequencing based HLA genotyping of the MS cohort resolved to at least the 4-digit level was performed as previously described using heterozygous ambiguity resolving primers where applicable [27,28].

### EBV amplification

The N- and C-terminal ends excluding the glycine-alanine rich regions of the EBNA-1 gene were amplified using semi-nested PCRs and fully automated setup utilising Biomek FX robots. EBV reference strain B95-8 was extracted from a B95-8 transformed cell line and was diluted and used as a control in each EBV PCR. All PCR reactions were performed using Roche High Fidelity Taq in 25μl reactions with forward and reverse primers at a concentration of 25pmol/μl. All primers used for amplification have been previously published and named according to the position in the reference strain B95-8 [11,45,46], as summarised below:

107754F: TCCGGGCTGCGAGTAATTGG
107881F: GTCTGCACTCCCTGTATTCA
109111F: TCATCATCATCCGGGTCTCCACCGC
108160R: GGACACCATCTCTATGTCTTGGCC
109135R: GCGGTGGAGACCCGGATGATGATGA
109459R: CCCAAGTTCCTTCGTCGGTAGTCC
109759R: CTCCATCGTCAAAGCTGCA
109869R: CTGCCCTTCCTCACCCTCAT
109970R: CAACAGCACGCATGATGTCT

For amplification of the N-terminal end the first round primer pair 107754F-109135R (PCR1) resulting in a 1381 base pair fragment (bp) was used. Detailed information about size and location of EBNA-1 PCRs with reference to the EBV strain B95-8 can be found in S1 Fig. Second round amplification was then performed using either 107881F-109135R (PCR2) or 107754F-108160R (PCR3), resulting in 1254bp or 406bp fragments respectively. C-terminal EBNA-1 PCR was performed as described previously [27]. Briefly, first round amplification with the primer pair EBV109111F-EBV109970R (PCR 4) resulted in an 859bp fragment. A semi-nested PCR was followed using the primer combination 109111F-109869R (PCR5) resulting in a final 758 base pair product. Alternatively, shorter semi-nested PCRs were performed using the primer combinations 109111F-109759R (PCR6, 648bp) and 109111F-109459R (PCR7, 348bp) respectively. In some cases PCRs with alternative primers have been performed. For an overview of primer pairs used in each PCR, nucleotide coordinates of primers within the B95-8 reference strain as well as primer melting temperatures, elongation times and product sizessee S1 Table. Successful PCR samples were purified using magnetic particles with AMPure (Beckman Coulter) on Biomek FX robots and stored at 4°C until further use.

### Sanger sequencing and analysis

Samples were directly sequenced on an automated 96 capillary ABI 373 DNA Sequencer, followed by purification of sequencing products with magnetic particles using CleanSEQ (Beckman Coulter) on Biomek FX robots. Analysis of electropherograms was performed using the ASSIGN V4.0.1.36 software (Conexio Genomics). Threshold for mixture detection in Sanger sequencing has been established to be ~30%. For construction of the Phylogenetic tree, 53 MS sequences and reference strains B95-8, AG876, GD1 and HKNPC1 covering the majority of nucleotide positions B95-8: 109135–109815 (EBNA-1: 1186–1866) were included. Genetic distance was visualized using the Neighbour-joining method based on the p-distance model with pairwise deletion within the PHYLIP (Phylogeny Inference Package) version 3.695 [47].

### 454 FLX sequencing

For the 454 FLX sequencing strategy, 24 samples were pooled in a single FLX lane. 20 MS samples were selected based on successful Sanger sequencing for three epitopes of interest (EBNA-1 aa PPP: 401–416, AEG: 482–496 and MVF: 563–577). Three additional MS samples were included that displayed a band of correct size on a 1% agarose gel but were not successfully sequenced by Sanger methods. The B95-8 strain of EBV was used as a control in each EBV PCR and was also selected as a control for FLX sequencing.

First round C-terminal EBV PCRs products with the primer combination EBV109111F-EBV109970R (PCR4) were used as templates to generate shorter second round PCRs for the selected deep sequencing samples. For PCR length and location please refer to S1 Fig. This nested PCR was performed with molecular barcoded primers. These tags consisted of eleven nucleotides unique extension to the 5’ end of the second round primers EBV109111F and EBV109869R (PCR5b) resulting in 780bp amplicons. This method facilitates sample multiplexing while also increasing the ability to accurately assign reads back to the sample. Resulting PCR amplicons were pooled at equimolar ratios (3x10^11^ copies each) to achieve similar number of reads. Standard Library was constructed using 454 Roche Titanium Chemistry protocol. The denatured DNA library was immobilized onto beads and emulsified with the amplification reagents in a water-in-oil mixture and clonally amplified (emPCR). Following emPCR, the capture beads with bound DNA were enriched according to the 454FLX titanium manual and used for pyrosequencing on one lane of an eight lane 454 FLX sequencing run according to the 454 sequencing manual.

The reads obtained from the sequencing were separated according to the unique tags and linked back to the original samples, using the Next*GENe* software package from SoftGenetics, Inc (State College, PA, USA). Further analysis was performed with inhouse software.

### FLX data analysis

The Next*GENe* software package version 2.3.0 (SoftGenetics, Inc, USA) was used to create a consensus sequence present at >45% for each sample based on the B95-8 EBV reference strain (GenBank accession number: V01555.2). For each sample, all reads were aligned to the consensus using a pairwise alignment. The pairwise alignments were combined into a multiple alignment by matching the reference positions for all aligned pairs. The aligned files were used to detect homopolymers, which are known to occur in FLX sequencing as artefacts. During analysis, all homopolymers not present in the respective consensus sequence have been excluded. Minorities present at <1% were not taken into consideration and insertion deletions were also excluded except for a strain specific in-frame insertion of three amino acids (glycine (GGA), aspartic acid (GAT), aspartic acid (GAC)) at position 2367 of EBNA-1 position (109818/109819 of reference strain B95-8) in several samples, which has been described previously [11]. All mutations detected were additionally manually analysed in the raw FLX data file of each sample to exclude any contribution of homopolymer errors. A nucleotide change was considered a real mutation if the mutation could not have been caused by a nucleotide insertion/deletion before or after the mutation. Additionally, mutations were only taken into consideration if they were present in at least three sequences, independent of the total number of individual reads per sample. Mutations within three nucleotides from the beginning or end of a read were excluded. Furthermore, unresolved nucleotide mixtures within reads were few, but indicated low signal quality in this position and were not taken into consideration.

### Epitope predictions

HLA binding algorithms (NetMHCII, NetMHCIIpan) were utilized to identify potential HLA-DRB1*1501 class II HLA epitopes within the EBNA-1 Sanger derived sequences. Additionally, predictions were performed for two known HLA-DRB1*1501 class II EBNA-1 epitopes (denoted AEG and MVF) using all FLX consensus and Sanger sequences generated. All predicted 15mer peptides which resulted in HLA-DRB1*1501 strong (<50 nM) and weak binders (50–500 nM) were selected and tested for potential cross-reactivity on a dataset of CNS proteins enriched for axoglial proteins (human protein reference database (HPRD.org) and [42], as well as a selection of brain proteins derived from NCBI (S2 Table). Amongst these potential cross-reactive epitopes, a subset of epitopes sharing the majority of peptide amino acid residues within the epitope core HLA-binding sequence of nine amino acids, were identified.

### Statistical analysis

We assessed whether EBNA-1 nucleotide polymorphism at each position was significantly associated with MS risk alleles by grouping alleles according to previous genetic analysis by our group [27]. Samples were categorized as carrying high MS risk, low MS risk or neutral risk alleles, respectively, if they carried at least one risk allele (HLA-DR1*08, *15, *16); at least one protective allele (HLA-DR1*04, *07, *09) and no risk allele; or two neutral risk alleles. Tables of nucleotide frequencies by risk groups were created at each nucleotide position and associations assessed by Fisher exact tests. We assessed clustering based on all 62 C-terminal Sanger sequences using the “partitioning around medoids” (PAM) method [48]. All nucleotide positions demonstrating some nucleotide variation were included and clusters displayed via a plot of the first two principal components [48]. Associations of HLA risk groups with viral clusters found in the cluster analysis were also assessed using Fisher exact tests. Analyses were carried out using TIBCO Spotfire S+ 8.2 (Somerville, MA).

## Results

**Fig 1:**
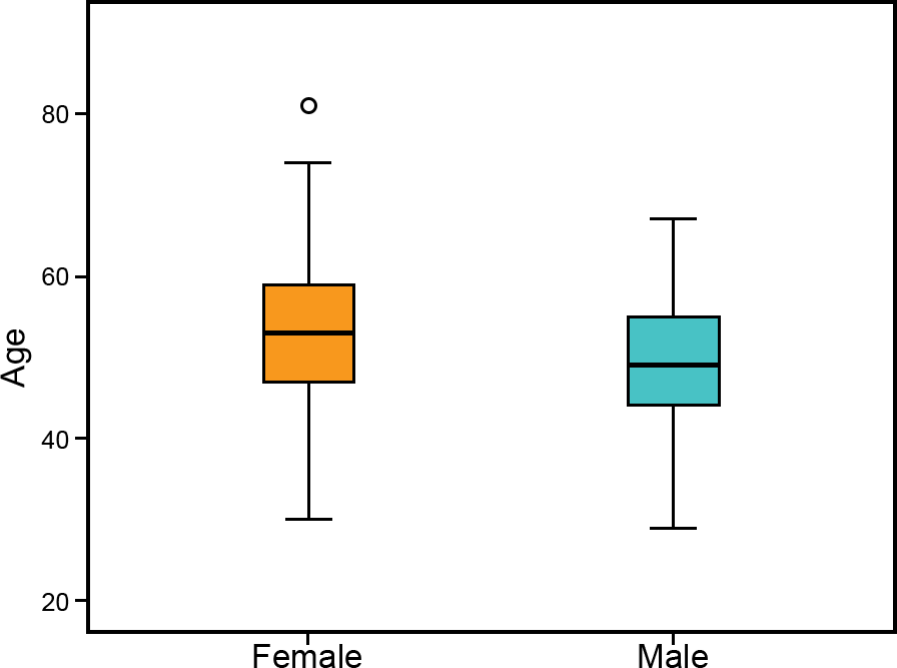
Age and gender distribution of patients recruited from the Perth Demyelinating Disease Database.

Epstein Barr Nuclear Antigen-1 (EBNA-1) was successfully amplified for bulk (Sanger) sequencing in 76 MS samples from DNA extracted buffy coats without any requirement for primary culture to enrich for EBV episomes. All samples were obtained from participants in the Perth Demyelinating Disease Database (PDDD) with confirmed MS, reflecting a wide range of age and disease severity (Fig 1). Females were more prevalent (73%) than males (27%) in the study population, with a slightly higher age (median: 53) compared to males (median: 49). Within this dataset, 62 samples were successfully sequenced across the EBNA-1 C-terminal region, which is known to contain the majority of MHC Class II-restricted T-cell epitopes [49–51], and a subset of 37 samples was sequenced in the N-terminal EBNA-1 region additionally. Sequences for both the N- and C-terminal end were obtained from 23 samples.

### Wild-type EBNA-1 sequence variation

**Fig 2:**
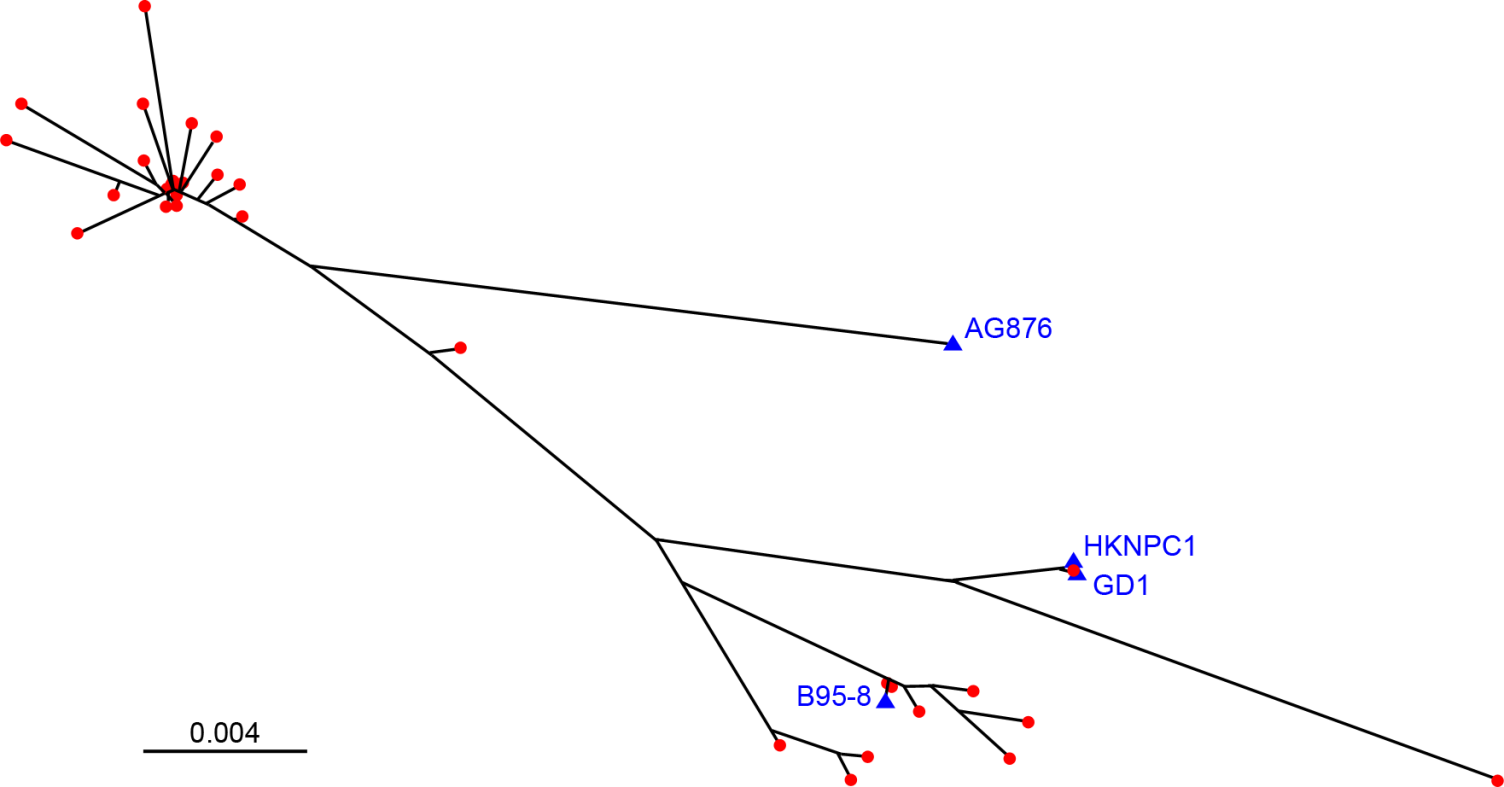
Phylogenetic tree of C-terminal EBNA-1 Sanger sequences including the reference strains B95-8, AG876, GD1 and HKNPC1. Phylogenetic tree covering nucleotide positions B95-8: 109135–109815; EBNA-1: 1186–1866; blue triangles: EBV reference strains; red dots: MS cases (n = 53).

The C-terminal region of EBNA-1 demonstrated sequence variation within four major clusters with additional minor variation (Fig 2). In keeping with previous analyses of wild-type EBNA-1 sequences [9,11], wild-type sequences showed strong similarity to the EBV reference strain B95-8 in only a minority of cases (9/53, 16.9%) and none clustered with the type 2 AG876 strain. No nucleotide mixtures were identified by Sanger sequencing methods that would indicate the presence of multiple EBNA-1 populations, either as a result of mutation or superinfection with multiple strains. N-terminal sequence analysis showed high conservation and identified only three different variants: 19 samples demonstrated 100% sequence similarity with the N terminal sequence of the EBV reference strain B95-8, while 12 sequences matched the previously identified type 2 EBV strain AG876 which differs to B95-8 in the positions: Q16E, E18G, D24E, S27G and A85T [52]. Three of these positions (Q16E, E18G, S27G) have previously been described to occur in combination (44). The third variant occurred in seven samples and aligned well with AG876 but contained two mismatches to it: EBNA-1 amino acid positions: V70A), (Q74P.

### Association of HLA and viral sequence variation

**Fig 3:**
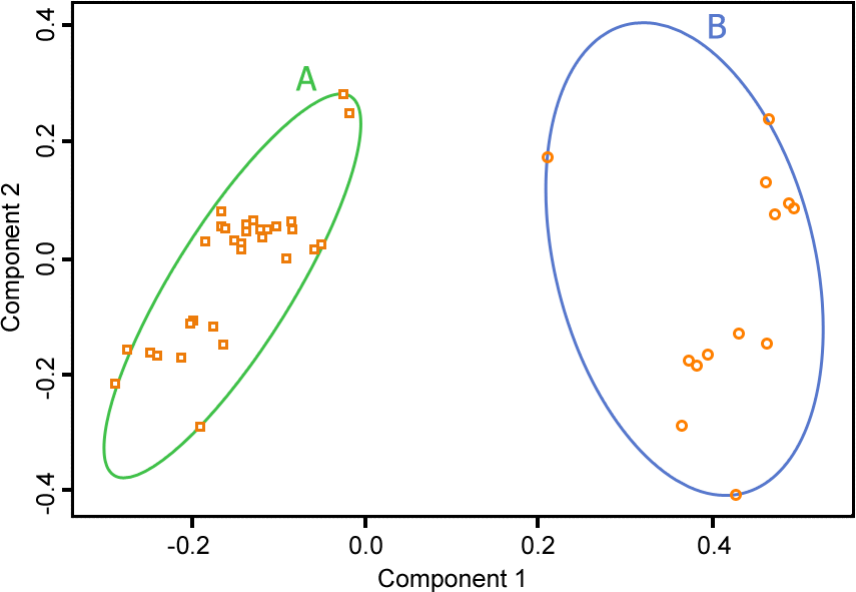
Principal Component plot (Component 1 vs Component 2) for cluster analysis based on the C-terminal EBNA-1 Sanger sequences revealed two distinct populations of 47 (A) and 15 (B) cases.

Association analysis of MS risk group with EBNA-1 sequence variation revealed nine positions at which individuals in the high risk group had nucleotide frequencies significantly differing from the other risk groups with p<0.05 –namely positions 1428 (P476), 1460 (A487), 1475, (S492), 1498 (D499), 1690 (M563), 1722 (V574), 1754 (T585), 1782 (R594) and 1785 (V595). Cluster analysis based on the C-terminal Sanger sequences described in Methods revealed two distinct populations (Fig 3), a main cluster (A) of 47 cases and a smaller cluster (B) of 15 cases. Across the nine positions with significant HLA association there were 373 consensus nucleotides and seven non-consensus among the 47 cases in cluster A and just 10 consensus nucleotides and 109 non-consensus in cluster B. Hence at these nine positions the two clusters were almost mutually exclusive.

When assessed for HLA MS risk group, cluster A contained 5, 9 and 33 individuals classified as MS risk neutral, protective and high, respectively, compared with the smaller cluster (B) which contained 4, 6 and 5 individuals with neutral, protective and high MS risk. The high MS risk group was thus significantly over-represented in the large cluster (p = 0.016).

### 454 deep sequencing

Additional sequencing of the C-terminal EBNA-1 fragment (EBNA-1 nucleotide positions 1160–1906) was then undertaken utilising FLX technology for 23 samples and a B95-8 control. Among these, 14 samples and the B95-8 control had coverage of all positions between 80–1632 reads, while 10 samples had very low coverage of reads per position (average reads <15) and were not included in subsequent sequence minority analysis. As shown in Table 1, low-level EBNA-1 sequence variation could be detected although only two samples (samples 1 and 5) showed the presence of minor sequence variants at a level of ≥10%. Mixtures occurred at nucleotide positions 1190 (R397) and 1588 (P529) in sample 1 and at 14 different positions in sample 5: 1286 (V429), 1420 (G473), 1421 (S474), 1428 (P476), 1460 (A487), 1475 (S492), 1498 (D499), 1561 (L520), 1572 (T524), 1660 (P553), 1690 (M563), 1722 (V574), 1754 (T585), 1782 (R594) and 1785 (V595). Silent mutations occurred at amino acid positions G473, D499, L520, P529 and P553, whereas the other mutations lead to a mixture of wild type and variant amino acids: R397R/G, V429V/M, S474S/T, P476P/Q, A487A/T, S492S/C, T524/TI, M563M/I, V574V/G, T585T/P, R594R/K and V595V/A. Lowering this threshold to 5% revealed minority variants in 3.6% positions (27/749 nucleotides sequenced); increasing to 5.0% at a 2% cut-off (37/749 nucleotides) and 8.3% at a 1% cut-off (62/749 nucleotides). As noted in Table 1, most of the mutations that defined minority variants were unique to individual samples (42 individual positions) (Table 1).

**Table 1:** EBNA-1 quasispecies detected with FLX sequencing. Minority EBV sequence variants at a level of ≥10% were detected in two samples only. Sequence mixtures present at a ≥5% threshold revealed minority variants in 3.6% of investigated nucleotide positions (27/749 nucleotides sequenced,), increasing to 5.0% at a ≥2% cut-off (37/749 nucleotides,) and 8.3% at a ≥1% cut-off (62/749 nucleotides,). Samples 7, 8 and 13 did not have minority species present at ≥1%.

EBNA-1 aa pos	397	**410**	411	422	425	429	431	432	435	435	459	**459**	460	469	**473**	474	476	**484**	487	492	**499**	507	508	**520**	524	525	525	528	528	**529**	533	**534**	536	539	**549**	**550**	**553**	**559**	**561**	563	574	585	594	595	595
B95-8 aa	R	G	E	G	G	V	P	G	E	E	R	R	K	R	G	S	P	G	A	S	D	V	F	L	T	A	A	I	I	P	L	T	L	L	P	Q	P	V	Y	M	V	T	R	V	V
B95-8 codon	AGG	GGG	GAA	GGC	GGT	GTG	CCG	GGA	GAG	GAG	CGC	CGC	AAA	CGT	GGT	TCC	CCG	GGT	GCT	AGT	GAC	GTG	TTC	CTA	ACT	GCC	GCC	ATT	ATT	CCA	CTT	ACA	TTG	CTC	CCA	CAA	CCG	GTC	TAT	ATG	GTT	ACA	AGG	GTG	GTG
EBNA-1 pos	1190	1231	1232	1265	1275	1286	1292	1296	1304	1305	1377	1378	1379	1406	1420	1421	1428	1453	1460	1475	1498	1520	1525	1561	1572	1574	1575	1583	1584	1588	1598	1603	1608	1616	1648	1651	1660	1678	1684	1690	1722	1754	1782	1785	1786
variant aa	G	G	Q	S	S	M	T	V	stop	G	H	R	Q	S	G	T	Q	G	T	C	D	M	L	L	I	$	$	$	T	P	I	T	S	I	P	Q	P	V	Y	I	G	P	K	A	A
variant codon	**G**GG	GG**A**	**C**AA	**A**GC	G**A**T	**A**TG	**A**CG	G**T**A	**T**AG	GG**G**	C**A**C	CG**A**	**C**AA	**A**GT	GG**C**	**A**CC	C**A**G	GG**C**	**A**CT	**T**GT	GA**T**	**A**TG	TT**A**	CT**C**	A**T**T	**SS**C	**SS**C	**RY**T	A**C**T	CC**G**	**A**TT	AC**G**	**T**CG	**A**TC	CC**G**	CA**G**	CC**A**	GT**A**	TA**C**	AT**T**	G**G**T	**C**CA	A**A**G	G**C**G	GC**T**
sample 1, 1%	AG										AG																			AG									AT					CT	
sample 1, 2%	AG																													AG									AT						
sample 1, 5%	AG																													AG															
sample 1, 10%	AG																													AG															
sample 2, 1%																										CG	CG					AG	CT												
sample 3, 1%										AG		AC	AC																							AG									
sample 3, 2%													AC																																
sample 3, 5%													AC																																
sample 4, 1%																		CT																											
sample 4, 2%																		CT																											
sample 5, 1%			CG			AG									CT	AT	AC		AG	AT	CT			AC	CT			AG			AC						AG			GT	GT	AC	AG	CT	
sample 5, 2%			CG			AG									CT	AT	AC		AG	AT	CT			AC	CT			AG			AC						AG			GT	GT	AC	AG	CT	
sample 5, 5%			CG			AG									CT	AT	AC		AG	AT	CT			AC	CT			AG			AC						AG			GT	GT	AC	AG	CT	
sample 5, 10%						AG									CT	AT	AC		AG	AT	CT			AC	CT												AG			GT	GT	AC	AG	CT	
sample 6, 1%																																			AG										
sample 10, 1%															CT																														
sample 11, 1%															CT																														
sample 12, 1%		AG				AG											AC		AG	AT	CT			AC	CT												AG			GT	GT	AC	AG	CT	
sample 12, 2%		AG				AG											AC		AG	AT	CT			AC	CT												AG			GT	GT	AC	AG	CT	

sample 12, 5%						AG											AC		AG		CT			AC																GT					
sample 14, 1%					AG																								CT																
B95-8, control 1%				AG			AC	GT	GT					AC								AG	AC											AC				AC							GT
B95-8, control, 2%				AG																																									

Subsequent capital letters indicate both nucleotide mixtures detected. Single capital letters indicate amino acids present. Amino acid positions representing silent mutations have been indicated in bold. EBNA-1 = Epstein-Barr virus nuclear antigen-1, aa = amino acid, pos = position

### Sequence conservation and relevance to HLA-DRB1*15 binding

As shown in Fig 3, EBNA-1 sequences were mapped against the standard reference strain B95-8 in order to illustrate known and putative HLA-DRB1*15-restricted epitopes and their relationship with EBNA-1 sequence conservation at the amino acid level. This approach, which utilises the ConSeq server [53], reflects amino acid conservation in terms of the influence of physicochemical properties of amino acid substitution as well as the frequency of sequence variation. Hence, ‘dips’ in the conservation plot (highlighted in red) reflect sites of variation that are likely to influence protein structure. Above these plots, predicted HLA-DRB1*15 binding sites are denoted along with the core binding regions, derived from NetMHCIIPan analysis [41]. As shown, this approach revealed two known HLA-DRB1*1501 restricted epitopes (AEGLRALLARSHVER (‘AEG’: aa 481–496) and MVFLQTHIFAEVLKD (‘MVF’: aa 562–577), as well as two overlapping putative epitopes covering a region between positions 502–543. In contrast, no HLA-DRB1*15 epitopes were identified within the N-terminal region of EBNA-1. The most frequent sequences at these epitope sites were AEGLR**T**LLAR**C**HVER and **I**VFLQTHIFAE**G**LKD (differences to B95-8 reference underlined). Variant sequences within these epitope regions are described in Table 2, along with comparisons of HLA-DRB1*15 binding affinity. As noted, there are no wild-type EBNA-1 variants within these known epitopes that would be predicted to abrogate HLA-DRB1*15 binding completely, although further studies will be required to establish if minor variations in binding affinity could influence the nature of the T cell response, noting that a previous study has demonstrated both regulatory and effector EBNA-1-specific CD4^+^ T cells with identical epitope specificity [18]. Interestingly, eight of the nine polymorphic sites from the cluster analysis fell within our predicted HLA-DRB1 epitopes including two changes in the previously described ‘AEG’ and ‘MVF’ epitopes, respectively.

**Table 2:** EBNA-1 sequence variation identified with next-generation sequencing technology, and impact on HLA-DRB1*15 binding affinity within known epitopes. The two most frequent variants for the A) AEG epitope and B) MVF epitope are printed in bold, the most frequent variant is underlined. All epitopes are predicted to be weak binders (affinity 50nM-500nM).

**Table 2A**																	NetMHCII		NetMHCIIpan	
nt B95-8	gca	gaa	ggt	tta	aga	gct	ctc	ctg	gct	agg	agt	cac	gta	gaa	agg	peptide	core	affinity(nM)	core	affinity(nM)
aa B95-8	A	E	G	L	R	A	L	L	A	R	S	H	V	E	R	AEGLRALLARSHVER	RALLARSHV	197	LRALLARSH	63
	-	-	-	-	-	-	-	-	-	-	-	-	-	-	-	**AEGLRALLARSHVER**	RALLARSHV	197	LRALLARSH	63
	-	-	-	-	-	V	-	-	-	-	-	-	-	-	-	AEGLRVLLARSHVER	RVLLARSHV	87	LRVLLARSH	61
	-	-	-	-	K	-	-	-	-	-	-	-	-	-	-	AEGLKALLARSHVER	KALLARSHV	165	LKALLARSH	88
	-	-	-	-	-	T	-	-	-	-	C	-	-	-	-	**AEGLRTLLARCHVER**	LRTLLARCH	267	LRTLLARCH	91
	-	-	-	-	-	A	-	-	-	-	S	-	-	-	-	AEGLRALLARSHVER	RALLARSHV	197	LRALLARSH	63
pos B95-8	482	483	484	485	486	487	488	489	490	491	492	493	494	495	496					
**Table 2B**																	NetMHCII		NetMHCIIpan	
nt B95-8	atg	gtc	ttt	tta	caa	act	cat	ata	ttt	gct	gag	gtt	ttg	aag	gat	peptide	core	affinity(nM)	Core	affinity(nM)
aa B95-8	M	V	F	L	Q	T	H	I	F	A	E	V	L	K	D	MVFLQTHIFAEVLKD	MVFLQTHIF	303	VFLQTHIFA	257
	-	-	-	-	-	-	-	-	-	-	-	-	-	-	-	**MVFLQTHIFAEVLKD**	MVFLQTHIF	303	VFLQTHIFA	257
	I	-	-	-	-	-	-	-	-	-	-	G	-	-	-	**IVFLQTHIFAEGLKD**	IVFLQTHIF	261	IVFLQTHIF	259
	-	-	-	-	-	-	-	-	-	-	-	-	F	-	-	MVFLQTHIFAEVFKD	VFLQTHIFA	281	VFLQTHIFA	270
	I	-	-	-	-	-	-	-	-	-	-	V	-	-	-	IVFLQTHIFAEVLKD	IVFLQTHIF	275	IVFLQTHIF	181
	M	-	-	-	-	-	-	-	-	-	-	G	-	-	-	MVFLQTHIFAEGLKD	VFLQTHIFA	272	MVFLQTHIF	386
	M	-	-	-	-	-	-	-	-	-	-	V	-	-	-	MVFLQTHIFAEVLKD	MVFLQTHIF	303	VFLQTHIFA	257
pos B95-8	563	564	565	566	567	568	569	570	571	572	573	574	575	576	577					

nt: nucleotides, aa: amino acid, pos: amino acid position, B95-8: wildtype EBV reference strain

### Identification of HLA-DRB1*15 epitopes within brain proteins homologous to EBNA-1

For this analysis we utilised three datasets enriched for CNS proteins [42], to identify candidate cross-reactive proteins that shared a propensity for HLA-DRB1*15 binding as well as homology to natural EBNA-1 sequences at these sites. This analysis was predicated on the hypothesis that (1) EBNA-1-specific T cell immunity reflects a standard model of HLA-restricted binding and antigen presentation, providing a long-term stimulus for T cell responses that could then (2) cross-react with CNS-specific antigens in a manner that requires HLA-restricted presentation but which may be less predictably associated with HLA binding affinity given the constraints of negative selection against high-affinity autoantigens, and the known altered topology of many autoreactive HLA-peptide-TCR interactions [54]. This approach is also informed by the previous demonstration of EBNA-1-specific CD4^+^ T cells capable of producing pro-inflammatory responses against myelin antigens in a seminal study by Lunemann and colleagues [33].

Thus, we initially selected EBNA-1 epitopes of interest based on HLA binding affinity, and then identified candidate CNS protein epitopes that would be predicted to bind HLA-DRB1*15 with sufficient affinity to allow antigen presentation (strong and weak binders with affinity threshold 500 nM) and which exhibited homology with the EBNA-1 epitope (threshold ≥3 residues within the 9 amino acid core binding region identified by NetMHCII and NetMHCIIPan analysis). Applying this approach to myelin proteins of known interest in MS research in the first instance reviewed in [55], we identified potential cross-reactive responses involving a number of major myelin antigens (Table 3) including experimentally confirmed HLA-DRB1*15-restricted epitopes associated with encephalitogenic T cell responses (asterisked) including 2',3'-cyclic-nucleotide 3'-phosphodiesterase, alpha B crystallin, myelin basic protein and oligodendrocyte-specific protein. Additionally, we identified several novel candidate antigens within glial fabrillary acidic protein, myelin proteolipid protein, neurofilament heavy polypeptide and myelin-oligodendrocyte glycoprotein.

**Table 3:** Putative HLA-DRB1*15 binders within autologous EBNA-1 peptide sequences and candidate myelin antigens.

Brain Peptides	EBNA-1 Peptides	Peptide Match Count	Brain Binding Scores	EBNA-1 Binding Scores	EBNA-1 Protein Position	Brain Protein Position	Brain Protein Accession No(s)
GKLYSLGNGRWMLTL	GSKTSLYNLRRGTTL	X…XX.X.X…XX	66	267	512	370	P09543
RGKLYSLGNGRWMLT	GGSKTSLYNLRRGVA	.X…XX.X.X....	41	347	511	369	P09543
LYSLGNGRWMLTLAK *	KTSLYNLRRGVALAI	..XX.X.X....XX.	306	45	514	372	P09543
SRGKLYSLGNGRWML *	SKTSLYNLRRGVALA	X…XX.X..X....	41	90	513	368	P09543
LSPFYLRPPSFLRAP **	TSLYNLRRGVALAIP	.X…XX....X..X	47	52	514	44	ACP18852
LSPFYLRPPSFLRAP **	TSLYNLRRGTALAIP	.X…XX....X..X	135	16	515	44	ACP18852
SPFYLRPPSFLRAPS	SLYNLRRGVALAIPQ	X…XX....X..X.	59	54	515	45	AAB23453
KTKEGVLYVGSKTRE	WVAGVFVYGGSKTSL	.......X.XXXX..	54	8	503	32	Q16143
EKTKEGVLYVGSKTR	NWVAGVLVYGGSKTS	........X.XXXX.	67	48	502	31	Q16143
TRLSLARMPPPLPTR	LRVLLARSHVERTTE	.X..XXX......X.	468	32	485	35	P14136
KLALDIEIATYRKLL	NLRRGIGLAIPQCLL	.X…X..X....XX	176	323	518	356	P14136
GKGRGLSLSRFSWGA	PQCRITPLSRLPFGM	…X…XXX…X.	287	384	528	131	AAC41944
EFAPVLLLESHCAAA ***	GLRVLLARSHVERTT	XX......XX....X	410	125	483	79	AAC41944
PGVLVLLAVLPVLLL	EGLRVLLARSHVERT	.X..XXXX…X…	301	8	483	153	Q16653
GPLVALIICYNWLHR	GPLRESIVCYFIVFL	XXX…X.XX.....	26	373	551	219	Q16653
GEGKVTLRIRNVRFS	AEGLRTLLARCHVXR	.XX..XX..X.....	89	16	482	106	Q16653
DPFYWVSPGVLVLLA †	QKFENIAXGLRTLLA	..X.....X…XXX	134	202	475	146	Q16653
DPFYWVSPGVLVLLA †	PKFENIAEGLKLLLA	..X.....X…XXX	185	438	475	146	Q16653
CSAVPVYIYFNTWTT	VAGVFVYGGXNTSLY	…X.XX…XX…	31	8	504	169	AAA59565
ATYNFAVLKLMGRGT	GTWVAGVLVYGGSKT	.X....XX…X..X	219	8	501	261	AAA59565
LLTFMIAATYNFAVL	LVMTKPAPTCNIKVT	X.....X.X.X..X.	70	32	582	254	AAA59565
EEITEYRRQLQARTT	ENIAEGLRVLLARSH	X.X.X..X.X.XX..	376	239	479	316	P12036
EMRGAVLRLGAARGQ	ENIAEGLRLLLARCH	X.....XXX..XX..	138	154	479	152	P12036
GAVLRLGAARGQLRL	AEGLRLLLARCHVER	…XXX..XX.....	67	88	482	155	P12036
TRLSFTSVGSITSGY	VAGVFVYGGSRTSLY	....X…XX.XX.X	206	71	504	398	P07196
KVVLIKNTLRSLEVL	KFENIAEGLRLLLAR	X…X…XX.X…	74	336	477	137	P23515
SLEVLNLSSNKLWTV	GLRVLLARSHVERTT	.X.XX…X....X.	62	125	484	147	P23515
PGTLINLTNLTHLYL	KTSLYNLRRGTALAI	…X.XX…X.X..	102	77	514	184	P23515
ENVSTTLRALAPRLM	ENIAEGLRALLARSH	XX....XXXX..X..	39	32	479	187	AAC25187
AGVLLILLALCALVA	AEGLRTLLARCHVER	X..X..XXX.X....	389	32	482	123	AAC25187
NVSTTLRALAPRLMR	NIAEGLRALLARSHV	X....XXXX..X…	25	16	480	188	AAC25187
CKPLVDILILPGYVQ ††	IKDLVMIKPAPTCNI	.X.XX.X…X....	48	120	578	65	AAC25187
ELEKAMVALIDVFHQ	EGLKALLARSHVERT	X..XX..X…X…	376	16	483	3	NP_006263
AFVAMVTTACHEFFE	VCYFMVFLQTHIFAE	....XX....X.X.X	457	71	558	76	NP_006263
FGAEILKKIPGRVST	NIAEGLKALLARSHV	..XX.XX....X…	51	16	480	91	NP_006746
GIRKFAADAVKLERM	NLRRGIALAVQQCRL	..X…X.XX…X.	387	32	519	311	NP_006746
PILAVLLFSSLVLSP	EGLRVLLARSHVERT	..X.XXX..X.X…	66	8	483	12	P25189
ILAVLLFSSLVLSPA	GLRVLLARSHVERTT	.X.XXX..X.X....	65	16	484	13	P25189
EFAPVLLLESHCAAA	EGLRVLLARSHVERT	X…XXX..XX....	177	8	483	410	P20916
VEFAPVLLLESHCAA	AEGLRVLLARSHVER	.X…XXX..XX…	250	16	482	409	P20916
PGVLVLLAVLPVLLL	EGLRVLLARSHVERT	.X..XXXX…X…	301	8	483	153	Q16653
VLGPLVALIICYNWL	NLRRGVALAIPQCRL	.X…XXX.X....X	148	32	519	217	Q16653
GAEIRHVLVTLGEKM	GTWVAGVLVYGGSKT	X.....XXX..X.X.	358	8	501	106	P60660
KLRRGDLPFVVPRRM	NLRRGIALAVQQCRL	.XXXX....X…X.	498	32	519	1920	P35579
AEELRARLTAKKQEL	AEGLRALLARSHVER	XX.XXX.X.....X.	466	115	482	899	P35579

Underlined is the core of the peptide. Binding score (nM) prediction using NetMHCII and NetMHCIIpan. Binding score <50: strong binder, binding score 50–500 weak binder.

*Known CNP epitope [56]

**Known aB-crystallin epitope [57]

***Known MBP epitope [58]

†Known MOG epitope [59]

††Known OSP epitope [60].

We then extended this analysis to a larger set of central nervous system antigens enriched for axoglial proteins that maintain myelinated nerves and nodes of Ranvier critical for saltatory conduction reviewed in [61], noting recent evidence that the axoglial apparatus may be targeted in the earliest phases of multiple sclerosis lesion development [62,63]. As described in Table 4, which presents a subset of results based on optimal EBNA-1 epitope binding and core match values ≥3, this analysis identified a larger set of potentially cross-reactive CNS proteins including neurofascin [62] as well as a number of other proteins involved in actin organisation and paranodal assembly like ankyrins, contactin-associated proteins as well as gelsolin [64].

**Table 4:** Extended axoglial brain protein dataset with HLA-DRB1*1501 predicted brain epitopes overlapping with predicted EBV binders.

Brain Peptides	EBNA-1 Peptides	Peptide Match Count	Brain Binding Scores	EBNA-1 Binding Scores	EBNA-1 Protein Position	Brain Protein Position	Brain Protein Accession No(s)	Brain Protein Genbank Description(s)
TGQFVYCGKKAQLNI	AGVFVYGGSKTSLYN	.X.XXX.X.X..X..	203	8	505	84	P62917	60S ribosomal protein L8
GQFVYCGKKAQLNIG	GVFVYGGSKTSLYNL	X.XXX.X.X..X…	237	8	506	85	P62917	
QFVYCGKKAQLNIGN	VFVYGGSKTSLYNLR	.XXX.X.X..X....	302	16	507	86	P62917	
GDRGKLARASGNYAT	GLRTLLARCHVERTT	X.X..XXX......X	329	32	484	121	P62917	
REEIHEYRRQLQART	FENIAEGLRVLLARS	.X.X.X..X.X.XX.	231	32	478	309	Q16352	Alpha-internexin
EEIHEYRRQLQARTI	ENIAEGLRTLLARCH	X.X.X..X.X.XX..	189	32	479	310	Q16352	
VAELLATLQASSQAA	IAEGLRTLLARSHVE	.XX.X.XX.X.X…	348	280	480	235	Q16352	
VASVLLEAGAAHSLA	VAGVLVYGGSKTSLY	XX.XX…X…XX.	140	22	504	545	Q01484	Ankyrin-2
DVASVLLEAGAAHSL	WVAGVLVYGGSKTSL	.XX.XX…X…XX	178	29	503	544	Q01484	
ELLLERGAPLLARTK	ENIAEGLRPLLARCH	X…X…XXXXX..	112	408	479	316	Q01484	
RITCRLVKPQKLSTP	RRGIGLAIPQCLLTP	X....X..XX.X.XX	227	369	520	948	AAA51732	
DIVKLLLPRGGSPHS	NWVAGVLVYGGSKTS	..X…X..XXX..X	74	48	502	583	AAA51732	
GAYVKLLSKTPELNL	GVLVYGGSKTSLYNL	X..X…XXX…XX	283	30	506	162	P27824	Calnexin
TAEAIKALGAKHCVK	IAEGLKALLARSHVE	.XX..XXX.X…X.	69	16	481	209	P30042	ES1 protein homolog, mitochondrial
AEAIKALGAKHCVKE	AEGLKALLARSHVER	XX..XXX.X…X..	73	16	482	210	P30042	
LSGESLGHLRSLGAL	GSKTSLYNLRRGVAL	.X..XX..XX…XX	406	16	512	189	P0C6S8	Leucine-rich repeat and immunoglobulin-like
SGESLGHLRSLGALR	SKTSLYNLRRGVALA	X..XX..XX…XX.	54	16	513	190	P0C6S8	
LSGESLGHLRSLGAL	GSKTSLYNLRRGIAL	.X..XX..XX…XX	406	16	512	189	P0C6S8	
AFLGLRQIRLLNLSN	VALAIPQCRLTPLSR	..X…X.XX..XX.	34	32	524	313	P0C6S8	
LGLRQIRLLNLSNNL	LAVQQCRLTPLSRLP	X…X.XX..XX…	26	32	526	315	P0C6S8	
AGVLLILLALCALVA	AEGLRTLLARCHVXR	X..X..XXX.X....	389	16	482	123	NP_005593	claudin-11 isoform 1
VLLILLALCALVATI	GLRTLLARCHVERTT	.X..XXX.X....X.	349	32	484	125	NP_005593	
LYCIYVAIGQKRSTV	RRGIALAIPQCRLTP	…X..XX.X.X.X.	124	32	521	242	P25705	ATP synthase subunit alpha, mitochondrial
SPGWLADGSVRYPIV	QPGPLRESIVCYFIV	.XX.X....X.X.XX	398	361	550	302	Q96GW7	Brevican core protein
LLGRWKALLIPPSSP	NLRRGIALAIPQCXL	.X.X..XX.XX....	78	32	519	893	Q96GW7	
EDSLECLRAMLSANI	ENIAEGLRALLARSH	X…X.XXX.X....	105	32	479	661	Q00610	Clathrin heavy chain 1
SLECLRAMLSANIRQ	SLYNLRRGISLAIPQ	XX..XX…X..X.X	38	77	516	663	Q00610	
GVLLILLALCALVAT	EGLRTLLARCHVERT	..X..XXX.X....X	387	32	483	124	O75508	Claudin-11
VLLILLALCALVATI	GLRTLLARCHVERTT	.X..XXX.X....X.	349	32	484	125	O75508	
ENLIVPGGVKTIEAN	AGVFVYGGSKTSLYN	....X.XX.XX…X	318	8	505	47	Q14194	Dihydropyrimidinase-related protein 1
DNLIVPGGVKTIEAN	AGVLVYGGSKTSLYN	....X.XX.XX…X	351	24	505	47	Q14195	
ELRREISYAIKNIHG	NLRRGIALAIPQCXL	.XXX.X..XX.....	74	32	519	383	Q05193	Dynamin-1
KELRREISYAIKNIH	YNLRRGISLAIPQCR	..XXX.XX.XX....	116	237	518	382	Q05193	
DEKELRREISYAIKN	SLYNLRRGISLAIPQ	....XXX.XX.XX..	246	77	516	380	Q05193	
EDLRRGLVMVKPGSI	KDAIKDLVMTKPAPT	.X....XXX.XX…	162	32	576	332	P49411	Elongation factor Tu, mitochondrial
DLRRGLVMVKPGSIK	DGIKDLVMTKPAPTC	X....XXX.XX....	93	32	577	333	P49411	
IVFRGEHGFIGCRKV	IVFLQTHIFAEGLKX	XXX…X.X....X.	233	16	563	386	Q16658	Fascin
IVFRGEHGFIGCRKV	IVFLQTHIFXEGLKD	XXX…X.X....X.	233	32	563	386	Q16658	
TGAQELLRVLRAQPV	ENIAEGLRVLLARSH	....X.XXXX.X…	52	32	479	616	P06396	Gelsolin
GDSYIILYNYRHGGR	GGSKTSLYNLRRGVA	X.X…XXX.X.X..	11	347	511	471	P06396	
KVKAHGKKVLGAFSD	ENIAEGLRVLLARSH	…X.X..XX.X.X.	498	32	479	60	P68871	Hemoglobin subunit beta
AFLGLRQIRLLNLSN	VALAIPQCRLTPLSR	..X…X.XX..XX.	34	32	524	313	P0C6S8	Leucine-rich repeat and immunoglobulin-like
LGLRQIRLLNLSNNL	LAVQQCRLTPLSRLP	X…X.XX..XX…	26	32	526	315	P0C6S8	
YSWGMAVNVYSTSIT	GNWVAGVLVYGGSKT	..X…X.XX..X.X	239	8	501	40	Q15555	Microtubule-associated protein RP/EB family member 2
WGMAVNVYSTSITQE	WVAGVFVYGGSKTSL	X…X.XX..X.X..	252	8	503	42	Q15555	
LPRWQLALAVGAPLL	NLRRGIALAVQQCRL	..X…XXXX....X	97	32	519	36	O94826	Mitochondrial import receptor subunit TOM70
IFQKLMFKNAPTPQE	IKDLVMTKPAPTCNI	X....X.X.XXX…	90	16	579	114	P13591	Neural cell adhesion molecule 1
VKIFQKLMFKNAPTP	DAIKDLVMTKPAPTC	..X....X.X.XXX.	40	32	577	112	P13591	
SLLVTRLQKALGVRQ	SLYNLRRGIALAVQQ	XX…X…XX.X.X	121	57	516	5	Q99798	Aconitate hydratase, mitochondrial
SLLVTRLQKALGVRQ	SLYNLRRGIALAVSQ	XX…X…XX.X.X	121	58	516	5	Q99798	
SLLVTRLQKALGVRQ	SLYNLRRGIALAVPQ	XX…X…XX.X.X	121	72	516	5	Q99798	Aconitate hydratase, mitochondrial
NKGIGLAIVRDLCRL	RRGIGLAIPQCLLTP	..XXXXXX…X…	436	369	521	14	P16152	Carbonyl reductase NADPH 1
RRGRLAVSFRFRTWD	LRALLARSHVERTTD	.X..XX.X…XX.X	77	407	485	379	P78357	Contactin-associated protein 1
LGAALRRCAVAATTR	LRLLLARCHVERTTE	X…X.XX.X..XX.	401	277	485	2	P20674	Cytochrome c oxidase subunit 5A, mitochondrial
LGAALRRCAVAATTR	LRTLLXRCHVERTTX	X…X.XX.X..XX.	401	248	484	2	P20674	
GTRLSLARMPPPLPT	GLRLLLARCHVERTT	X.XX.XXX......X	326	139	484	34	P14136	Glial fibrillary acidic protein
VVFFNVPEKLRLPDA	QKFENIAEGLRLLLA	..X.X..X.XXX..X	416	382	476	134	P78559	Microtubule-associated protein 1A
TAYARLRGIEQAVQS	SLYNLRRGIALAVQQ	..X…XXX..XXX.	482	57	516	541	Q16891	Mitochondrial inner membrane protein
NTAYARLRGIEQAVQ	TSLYNLRRGIALAVQ	…X…XXX..XXX	349	63	515	540	Q16891	
FTLKVLTTRGVAERT	EGLRVLLARSHVERT	..X.XX..X…XXX	209	113	483	229	O94856	Neurofascin
TLKVLTTRGVAERTP	GLKLLLARSHVERTT	.XX.X..X…XXX.	368	96	484	230	O94856	
FTLKVLTTRGVAERT	EGLKLLLARSHVERT	..XX.X..X…XXX	209	87	483	229	O94856	
PTEIIAFSNRAEDFR	KFENIADSLRALLAR	..X.XX.X.XX…X	83	436	477	52	P32119	Peroxiredoxin-2
EIIAFSNRAEDFRKL	ENIADSLRALLARSH	X.XX.X.XX…X..	233	194	479	54	P32119	
GSVILLENLRFHVEE	GSKTSLYNLRRGVAL	XX…X.XXX..X..	33	119	512	114	P00558	Phosphoglycerate kinase 1
AGSVILLENLRFHVE	GGSKTSLYNLRRGVA	.XX…X.XXX..X.	26	347	511	113	P00558	
ASLQRVRRPVAMVMP	TSLYNLRRGVALAIP	.XX…XX.XX…X	268	52	515	95	Q15149	Plectin
LKKGLLSAEVARLLL	SQKFENIAEGLRLLL	..X....XX..XXXX	70	465	475	3847	Q15149	
VRVFRIFKLSRHSKG	VPQCRITPLSRLPFG	X…XX..XXX…X	13	475	528	299	P16389	Potassium voltage-gated channel subfamily A member 2
VRVFRIFKLSRHSKG	VSQCRITPLSRLPFG	X…XX..XXX…X	13	427	528	299	P16389	
LFPGVALLLAAARLA	LRRGVALAIPQCRLT	X..XXXX.....XX.	131	413	520	8	P30101	Protein disulfide-isomerase A3
ALFPGVALLLAAARL	NLRRGVALAIPQCRL	.X..XXXX.....XX	242	333	519	7	P30101	
PAIRLLYAKRPGIGL	GSRTSLYNLRRGIGL	.....XX..X.XXXX	40	250	512	190	O75061	Putative tyrosine-protein phosphatase auxilin
RLLYAKRPGIGLSPS	TSLYNLRRGIALAVS	..XX..X.XX.X..X	466	64	515	193	O75061	
SFASDPILYRPVAVA	SKTSLYNLRRGVALA	X..X…X.X.XX.X	253	90	513	97	P14618	Pyruvate kinase PKM
VEGSFVYKGGKIYKV	VAGVFVYGGSKTSLY	X.X.XXX.X.X....	120	100	504	105	P31150	Rab GDP dissociation inhibitor alpha
ARKKKLLEAQSHFRK	AEGLKLLLARSHVER	X…XXX.X.XX…	212	86	482	2074	Q13813	Spectrin alpha chain, non-erythrocytic 1
TTCIELGKSLLARKH	ENIAEGLKLLLARSH	....X..X.XXXX.X	62	254	479	1968	Q01082	

Underlined is the core of the peptide. Binding score (nM) prediction using NetMHCII and NetMHCIIpan. Binding score <50: strong binder, binding score 50–500 weak binder.

## Discussion

In this study we have proven the feasibility of obtaining EBNA-1 sequences directly from buffy coat samples, without any requirement for primary cultures that could theoretically be associated with preferential selection of viral sequence variants through *ex vivo* expansion. In this regard our findings are in keeping with those of Burrows and colleagues [11], who demonstrated similar patterns of EBNA-1 sequence variation predominantly within the C-terminal region in both MS cases and controls, in a study that did involve primary B lymphocyte cultures. Both studies, as well as a more recent analysis of spontaneously outgrown human lymphoblastoid cell lines [65] are in agreement in demonstrating that the majority of autologous sequences do not align closely with the widely used B95-8 laboratory strain–a result that is perhaps not surprising given that this strain was originally identified following transfusion-associated EBV in an elderly woman and subsequently selected for its ability to efficiently immortalise B lymphocytes [66].

**Fig 4:**
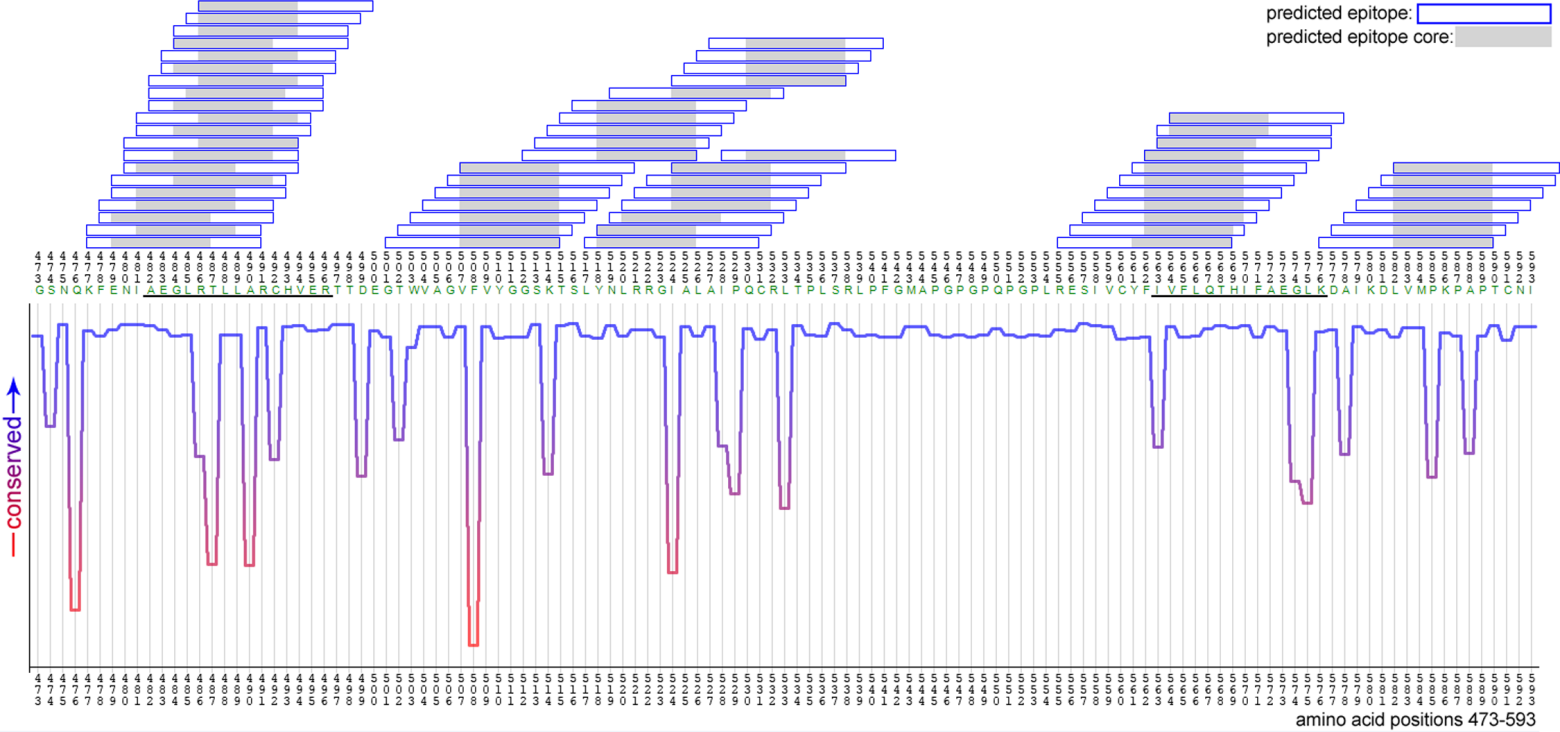
EBNA-1 sequence conservation and relationship to NetMHCII predicted HLA-DRB1*DR15-restricted epitopes derived from patient MS sequences. Shown are EBNA-1 amino acid positions 473–593. Predicted epitopes: blue boxes, known epitopes: black underlined, predicted epitope core: grey shade.

Our results are also in agreement with other studies that have identified similar patterns of EBNA-1 [67] and EBNA-2 [68] sequence variation when comparing MS cases and controls, albeit at low resolution in these cases, suggesting that MS susceptibility is not likely to be readily explained by an ‘encephalitogenic strain’ of EBV. We have also explored sequence variation within individual samples using next-generation sequencing techniques, to investigate if the presence of multiple viral sequence variants could indicate sites of immune selection pressure–as has been suggested previously for EBNA-1 [11]–and/or that infection with multiple EBV strains could represent a risk factor for MS disease as has been previously proposed [69]. We identified nucleotide mixtures present at 10 percent in two samples. Mixtures were primarily caused by point mutations leading to amino acid changes in 12 different positions compared to silent mutations in five positions only. This could indicate EBV superinfection or viral immune escape. While we were able to identify low-level EBNA-1 sequence variation in these samples (involving 8.3% of nucleotides at a 1% threshold), our results do not support a strong influence of intraindividual EBV sequence variation in MS disease risk and we cannot exclude that some of these point mutations are due to technical artefacts. It is however interesting to note that EBNA-1 sequence conservation described in Fig 4 (reflecting genetic variation as well as the impact this has on amino acid properties), does appear to map to HLA-DRB1 binding regions, although we were unable to identify natural sequence variants that were associated with abrogation of HLA binding using the NetMHCIIPan prediction algorithm. However, in the HLA-viral sequence variation association analyses, we could identify eight out of nine EBNA-1 polymorphic nucleotide positions significantly associated with MS risk alleles within these HLA-DRB1 binding regions, including two in the previously described HLA-DRB1*15 ‘AEG’ and ‘MVF’ epitopes respectively, noting in each case that the more common (wild-type) viral sequence was favoured in the presence of disease-associated HLA-DR alleles. These differences will be explored further in terms of their impact on epitope-specific CD4^+^ T-cell immune responses, acknowledging in relation to MS pathogenesis that important differences may relate to the selection of regulatory versus effector EBNA-1-specific CD4+ T cells [18], rather than simply reflecting immune evasion.

We have also explored the potential for autoantigens to be selected by cross-reactive EBNA-1-specific T cells, according to a shared propensity for HLA-DRB1*15-restricted antigen presentation as well as evidence of sequence homology. This concept is in keeping with previous experimentally-proven examples of this phenomenon (albeit without identification of specific epitopes involved) [35], with additional support from observations that HLA-DRB1*15-restricted immune responses are characterised by a relatively high level of TCR degeneracy that would favour cross-reactivity [70]. These results are preliminary and require experimental confirmation of their functional validity.The main purpose of this analysis was to create a platform for experimental design that acknowledges natural patient derived EBNA-1 sequence variants as the basis for epitope selection, while also expanding the possibilities of identifying novel candidate CNS antigens that may have a role in MS pathogenesis. As noted by Ben-Nun and colleagues [55] and Lassmann and colleagues [71], MS research is increasingly moving away from reductionist experimental models towards an interest in a wide array of myelin and axoglial antigen targets, which would be in keeping with a model of MS pathogenesis that allows for cross-reactive T cell (as well as humoral) responses that are initially driven by viral-specific responses–with EBNA-1 representing a legitimate candidate target based on previous work [27, 31–39].

These observations, along with continuing evidence of patient-specific heterogeneity of MS lesion pathology [72] and oligoclonal TCR repertoire [73] would support a model of MS disease pathogenesis in which virus-specific immunity, which is oligoclonal in nature as determined by viral sequence variation seen in this and other studies [9–11] as well as by polymorphic HLA-restricted antigen presentation, could then trigger cross-reactive autoimmune responses. We now hope to investigate these possibilities further, with a particular focus on the roles of both antigen-presenting B cells as well as antigen-specific T cells in provoking inflammatory immune responses. In this respect, we would anticipate that targeted T-cell immunotherapy is likely to require a patient-specific approach as recently performed by Pender and colleagues [37], while targeting EBV-infected B cells may have the potential to provide a more universal treatment strategy, particularly in light of recent evidence that antigen-experienced B cells within the central nervous system in MS cases are likely to be derived from the peripheral blood and lymph nodes [74,75].

## Supporting Information

S1 Fig Location of EBNA-1 PCR primers given with reference to nucleotide position of the B95-8 strain and PCR size given as base pairs (bp). Start and stop indicate the EBNA-1 gene. Purple indicates position of known epitopes.
(TIF)

S1 Table Primers used for EBV amplification. (DOC)

S2 Table Human brain proteins included in the analysis from NCBI database. (DOC)

S3 Table Sanger sequence Genbank Accession numbers. (DOC)
