# Supplementary figures and images for: Identifying Patient-Specific Epstein-Barr Nuclear Antigen-1 Genetic Variation and Potential Autoreactive Targets Relevant to Multiple Sclerosis Pathogenesis
Source: PLoS One. 2016 Feb 5;11(2):e0147567. doi: 10.1371/journal.pone.0147567 (PMC4744032; doi:10.1371/journal.pone.0147567)

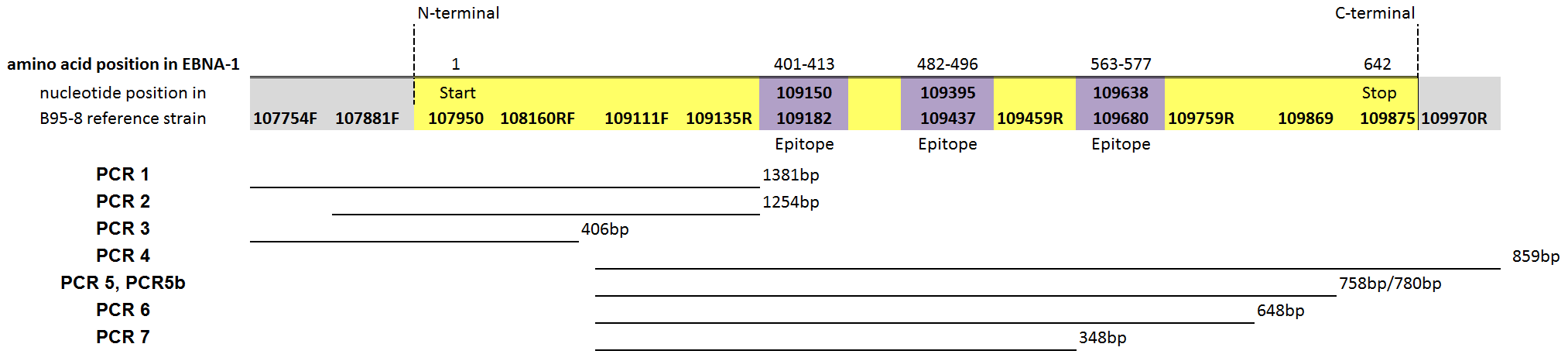

Supplement: S1 Fig — Start and stop indicate the EBNA-1 gene. Purple indicates position of known epitopes. (TIF) [file pone.0147567.s001.tif]
