# Supplementary material for: Identifying Patient-Specific Epstein-Barr Nuclear Antigen-1 Genetic Variation and Potential Autoreactive Targets Relevant to Multiple Sclerosis Pathogenesis
Source: PLoS One. 2016 Feb 5;11(2):e0147567. doi: 10.1371/journal.pone.0147567 (PMC4744032; doi:10.1371/journal.pone.0147567)
Supplement: S1 Table — (DOC) [file pone.0147567.s002.doc]

**S1 Table:** **Primers used for EBV amplification.** Number = coordinates within B95-8 reference strain, named for forward primers 3’ position, for reverse primers 5’ position of primer. *This PCR was also used for FLX sequencing. **PCR5 was also used for FLX sequencing with tagged primers = PCR5b. ^$^Primer binding site outside the EBNA-1 gene.

| **Forward primer** | **Reverse primer** | **PCR** | **Location within EBNA-1** | **PCR size (nt)** | **T (⁰C)** | **elongation (s)** |
| --- | --- | --- | --- | --- | --- | --- |
| 107754F^$^ | 109135R | PCR1 | N-terminus | 1381 | 59 | 100 |
| 107881F^$^ | 109135R | PCR2 | N-terminus | 1254 | 59 | 100 |
| 107754F^$^ | 108160R | PCR3 | N-terminus | 406 | 58 | 60 |
| 109111F | 109970R^$^ | PCR4* | C-terminus | 859 | 59 | 55 |
| 109111F | 109869R | PCR5** | C-terminus | 758 | 59 | 55 |
| 109111F | 109759R | PCR6 | C-terminus | 648 | 59 | 45 |
| 109111F | 109459R | PCR7 | C-terminus | 348 | 61 | 60 |

nt: nucleotide, T(⁰C): primer melting temperature in degree Celsius, s: seconds, EBNA-1: Epstein-Barr virus nuclear antigen-1
