# Supplementary material for: Identifying Patient-Specific Epstein-Barr Nuclear Antigen-1 Genetic Variation and Potential Autoreactive Targets Relevant to Multiple Sclerosis Pathogenesis
Source: PLoS One. 2016 Feb 5;11(2):e0147567. doi: 10.1371/journal.pone.0147567 (PMC4744032; doi:10.1371/journal.pone.0147567)
Supplement: S2 Table — (DOC) [file pone.0147567.s003.doc]

**S2 Table: Human brain proteins included in the analysis from NCBI database.** Given are accession numbers and description of brain derived proteins used for analysis.

| **Brain Protein Accession No(s)** | **Brain Protein Genbank Description(s)** | **Brain Protein Accession No(s)** | **Brain Protein Genbank Description(s)** |
| --- | --- | --- | --- |
| AAC41944.1 | Myelin basic protein | BAD96383 | Oligodendrocyte myelin glycoprotein variant, partial |
| AAC41944.2 | Myelin basic protein | CAA40684 | Oligodentrocyte-myelin glycoprotein |
| AAA59562.1 | Myelin basic protein | 1704256A | Oligodendrocyte myelin glycoprotein |
| NP_001020252 | Myelin basic protein isoform 1 | P55087 | Aquaporin-4 |
| NP_002376 | Myelin basic protein isoform 2 | AAH22286 | Aquaporin 4 |
| NP_001020261 | Myelin basic protein isoform 3 | AAB26957 | Aquaporin 4 |
| NP_001020263 | Myelin basic protein isoform 4 | P20916 | Myelin-associated glycoprotein |
| NP_002362 | Myelin and lymphocyte protein isoform a | NP_001186145 | Myelin-associated glycoprotein isoform c |
| AAI52452 | Myelin expression factor 2 | NP_542167.1 | Myelin-associated glycoprotein isoform b |
| AAV38349 | Myelin protein zero-like 1 | NP_002352 | Myelin-associated glycoprotein isoform a precursor |
| AAH17774 | Myelin protein zero-like 2 | AAB58805 | Myelin-associated glycoprotein |
| Q5T1S8 | Noncompact myelin-associated protein | NP_006263 | Protein S100-B |
| Q01453 | Peripheral myelin protein 22 | AAH01766 | S100 calcium binding protein B |
| NP_037411 | Myelin regulatory factor isoform 1 | P14136 | Glial fibrillary acidic protein |
| NP_001120864 | Myelin regulatory factor isoform 2 | P02511 | Alpha-crystallin B chain |
| NP_004526 | Myelin transcription factor 1 | AAB23453 | Alpha B-crystallin |
| AAB62700 | Myelin transcription factor 2 | ACP18852 | Alpha B-crystallin |
| P02689 | Myelin P2 protein | P04792 | Heat shock protein beta-1 |
| P25189 | Myelin protein P0 | P37837 | Transaldolase |
| P23515 | Oligodendrocyte-myelin glycoprotein | NP_006746 | Transaldolase |
| O75508 | Claudin-11/Oligodendrocyte-specific protein | P09543 | 2',3'-cyclic-nucleotide 3'-phosphodiesterase |
| AAC25187 | Oligodendrocyte-specific protein | NP_149124 | 2',3'-cyclic-nucleotide 3'-phosphodiesterase |
| NP_005593 | Claudin-11 isoform 1 | Q9NQC3 | Reticulon-4 |
| NP_001171985 | Claudin-11 isoform 2 precursor | NP_065393 | Reticulon-4 isoform A |
| NP_620450 | Oligodendrocyte transcription factor 1 | NP_722550 | Reticulon-4 isoform B |
| NP_005797 | Oligodendrocyte transcription factor 2 | NP_008939 | Reticulon-4 isoform C |
| P60201 | Myelin proteolipid protein | NP_997403NCBI | Reticulon-4 isoform D |
| AAA60350 | Proteolipid protein, partial | NP_997404 | Reticulon-4 isoform E |
| AAA60117 | Proteolipid protein | Q8TAK6 | Oligodendrocyte transcription factor 1 |
| NP_955772 | Myelin proteolipid protein isoform 2 | Q13516 | Oligodendrocyte transcription factor 2 |
| NP_002659 | Proteolipid protein 2 | NP_786923 | Oligodendrocyte transcription factor 3 |
| NP_001122306 | Myelin proteolipid protein isoform 1 | Q13875 | Myelin-associated oligodendrocyte basic protein |
| NP_000524 | Myelin proteolipid protein isoform 1 | NP_001265251 | Myelin-associated oligodendrocyte basic protein isoform a |
| AAA59565 | Myelin proteolipid protein | NP_001265252 | Myelin-associated oligodendrocyte basic protein isoform b |
| AAF86288 | Myelin proteolipid protein-like protein | NP_891980 | Myelin-associated oligodendrocyte basic protein isoform c |
| Q16653 | Myelin-oligodendrocyte glycoprotein | CAA52617 | Myelin oligodendrocyte glycoprotein |
| BAD96383 | Oligodendrocyte myelin glycoprotein variant, partial | P0C6S8 | Leucine-rich repeat and immunoglobulin-like domain-containing nogo receptor-interacting protein 3 |
| CAA40684 | Oligodentrocyte-myelin glycoprotein | AAB58805 | Myelin-associated glycoprotein |
| 1704256A | Oligodendrocyte myelin glycoprotein | NP_006263 | Protein S100-B |
| P55087 | Aquaporin-4 | AAH01766 | S100 calcium binding protein B |
| AAH22286 | Aquaporin 4 | P14136 | Glial fibrillary acidic protein |
| AAB26957 | Aquaporin 4 | P02511 | Alpha-crystallin B chain |
| P20916 | Myelin-associated glycoprotein | AAB23453 | Alpha B-crystallin |
| NP_001186145 | Myelin-associated glycoprotein isoform c | ACP18852 | Alpha B-crystallin |
| NP_542167.1 | Myelin-associated glycoprotein isoform b | P04792 | Heat shock protein beta-1 |
| NP_002352 | Myelin-associated glycoprotein isoform a precursor | P37837 | Transaldolase |
| NP_001265251 | Myelin-associated oligodendrocyte basic protein isoform a | NP_006746 | Transaldolase |
| NP_001265252 | Myelin-associated oligodendrocyte basic protein isoform b | P09543 | 2',3'-cyclic-nucleotide 3'-phosphodiesterase |
| NP_891980 | Myelin-associated oligodendrocyte basic protein isoform c | NP_149124 | 2',3'-cyclic-nucleotide 3'-phosphodiesterase |
| CAA52617 | Myelin oligodendrocyte glycoprotein | Q9NQC3 | Reticulon-4 |
| P0C6S8 | Leucine-rich repeat and immunoglobulin-like domain-containing nogo receptor-interacting protein 3 | NP_065393 | Reticulon-4 isoform A |
| A1KXE4 | Myelin-associated neurite-outgrowth inhibitor | NP_722550 | Reticulon-4 isoform B |
| Q9UQB8 | Brain-specific angiogenesis inhibitor 1-associated protein 2 | NP_008939 | Reticulon-4 isoform C |
| Q16143 | Beta-synuclein | NP_997403NCBI | Reticulon-4 isoform D |
| NP_001139526 | Alpha-synuclein isoform NACP140 | NP_997404 | Reticulon-4 isoform E |
| P07196 | Neurofilament light polypeptide; | Q8TAK6 | Oligodendrocyte transcription factor 1 |
| P07197 | Neurofilament medium polypeptide | Q13516 | Oligodendrocyte transcription factor 2 |
| P12036 | Neurofilament heavy polypeptide | NP_786923 | Oligodendrocyte transcription factor 3 |
| Q16352 | Alpha-internexin | Q13875 | Myelin-associated oligodendrocyte basic protein |
| NP_001135952 | Neuronal regeneration-related protein isoform a | NP_963885 | Neuronal membrane glycoprotein M6-a isoform 1 |
| NP_001135946 | Neuronal regeneration-related protein isoform b | NP_001001996 | Neuronal membrane glycoprotein M6-b isoform 2 |
| NP_001135947 | Neuronal regeneration-related protein isoform c | NP_005269 | Neuronal membrane glycoprotein M6-b isoform 3 |
| NP_001001994 | Neuronal membrane glycoprotein M6-b isoform 4 | Q96PE5 | Oligodendrocytic myelin paranodal and inner loop protein |
| NP_776169 | Neuronal growth regulator 1 precursor | NP_000546 | Neuronal migration protein doublecortin isoform a |
| NP_001032209 | Neuronal cell adhesion molecule isoform A precursor | NP_835365 | Neuronal migration protein doublecortin isoform b |
| NP_005001 | Neuronal cell adhesion molecule isoform B precursor | NP_835366 | Neuronal migration protein doublecortin isoform c |
| Q86UE4 | Astrocyte elevated gene-1 protein | NP_057648 | Cell cycle exit and neuronal differentiation protein 1 |
| NP_006001 | Mesencephalic astrocyte-derived neurotrophic factor precursor | NP_002046 | Glial fibrillary acidic protein isoform 1 |
| P55145 | Mesencephalic astrocyte-derived neurotrophic factor | NP_001124491 | Glial fibrillary acidic protein isoform 2 |
| Q9P232 | Contactin-3 | NP_001229305 | Glial fibrillary acidic protein isoform 3 |
| AAA51732 | Ankyrin | AAB22581 | Glial fibrillary acidic protein |
| P04637 | Cellular tumor antigen p53 |  |  |
|  |  |  |  |
