# Supplementary material for: Identifying Patient-Specific Epstein-Barr Nuclear Antigen-1 Genetic Variation and Potential Autoreactive Targets Relevant to Multiple Sclerosis Pathogenesis
Source: PLoS One. 2016 Feb 5;11(2):e0147567. doi: 10.1371/journal.pone.0147567 (PMC4744032; doi:10.1371/journal.pone.0147567)
Supplement: S3 Table — (DOC) [file pone.0147567.s004.doc]

**S3 Table: Sanger sequence Genbank Accession numbers.** C: Indicating the C terminus of EBNA-1, N: indicating the N terminus of EBNA-1; identical numbers for N and C terminal Sanger Sequence names indicate they are derived from the same individual.

| isolate | Accession number | isolate | Accession number | isolate | Accession number |
| --- | --- | --- | --- | --- | --- |
| 57326C.sqn 57326C | KT875892 | 57326C.sqn 39048C | KT875922 | 57326N.sqn 38720N | KT875952 |
| 57326C.sqn 38623C | KT875893 | 57326C.sqn 38772C | KT875923 | 57326N.sqn 38751N | KT875953 |
| 57326C.sqn 38924C | KT875894 | 57326C.sqn 38835C | KT875924 | 57326N.sqn 38812N | KT875954 |
| 57326C.sqn 38595C | KT875895 | 57326C.sqn 38838C | KT875925 | 57326N.sqn 38931N | KT875955 |
| 57326C.sqn 38624C | KT875896 | 57326C.sqn 38843C | KT875926 | 57326N.sqn 38996N | KT875956 |
| 57326C.sqn 38940C | KT875897 | 57326C.sqn 38850C | KT875927 | 57326N.sqn 39064N | KT875957 |
| 57326C.sqn 39136C | KT875898 | 57326C.sqn 38695C | KT875928 | 57326N.sqn 39070N | KT875958 |
| 57326C.sqn 38592C | KT875899 | 57326C.sqn 38798C | KT875929 | 57326N.sqn 39532N | KT875959 |
| 57326C.sqn 38611C | KT875900 | 57326C.sqn 38719C | KT875930 | 57326N.sqn 38604N | KT875960 |
| 57326C.sqn 38680C | KT875901 | 57326C.sqn 38720C | KT875931 | 57326N.sqn 38633N | KT875961 |
| 57326C.sqn 38685C | KT875902 | 57326C.sqn 38761C | KT875932 | 57326N.sqn 38685N | KT875962 |
| 57326C.sqn 38690C | KT875903 | 57326C.sqn 38751C | KT875933 | 57326N.sqn 38761N | KT875963 |
| 57326C.sqn 38694C | KT875904 | 57326C.sqn 39130C | KT875934 | 57326N.sqn 38895N | KT875964 |
| 57326C.sqn 38905C | KT875905 | 57326C.sqn 39000C | KT875935 | 57326N.sqn 38897N | KT875965 |
| 57326C.sqn 38918C | KT875906 | 57326C.sqn 38700C | KT875936 | 57326N.sqn 38767N | KT875966 |
| 57326C.sqn 38920C | KT875907 | 57326C.sqn 38767C | KT875937 | 57326N.sqn 38908N | KT875967 |
| 57326C.sqn 38922C | KT875908 | 57326C.sqn 38604C | KT875938 | 57326N.sqn 38781N | KT875968 |
| 57326C.sqn 38975C | KT875909 | 57326C.sqn 38895C | KT875939 | 57326N.sqn 38963N | KT875969 |
| 57326C.sqn 38979C | KT875910 | 57326C.sqn 38781C | KT875940 | 57326N.sqn 39006N | KT875970 |
| 57326C.sqn 38989C | KT875911 | 57326C.sqn 38963C | KT875941 | 57326N.sqn 39036N | KT875971 |
| 57326C.sqn 38806C | KT875912 | 57326C.sqn 39082C | KT875942 | 57326N.sqn 38979N | KT875972 |
| 57326C.sqn 38811C | KT875913 | 57326C.sqn 38810C | KT875943 | 57326N.sqn 39043N | KT875973 |
| 57326C.sqn 38834C | KT875914 | 57326C.sqn 38916C | KT875944 | 57326N.sqn 39051N | KT875974 |
| 57326C.sqn 38873C | KT875915 | 57326N.sqn 57326N | KT875945 | 57326N.sqn 39039N | KT875975 |
| 57326C.sqn 38890C | KT875916 | 57326N.sqn 38624N | KT875946 | 57326N.sqn 39063N | KT875976 |
| 57326C.sqn 38898C | KT875917 | 57326N.sqn 39136N | KT875947 | 57326N.sqn 38592N | KT875977 |
| 57326C.sqn 38903C | KT875918 | 57326N.sqn 38611N | KT875948 | 57326N.sqn 38623N | KT875978 |
| 57326C.sqn 39070C | KT875919 | 57326N.sqn 38636N | KT875949 | 57326N.sqn 38876N | KT875979 |
| 57326C.sqn 38996C | KT875920 | 57326N.sqn 38663N | KT875950 | 57326N.sqn 38905N | KT875980 |
| 57326C.sqn 39036C | KT875921 | 57326N.sqn 38694N | KT875951 |  |  |
